# Behçet‘s Syndrome Apart From the Triple Symptom Complex: Vascular, Neurologic, Gastrointestinal, and Musculoskeletal Manifestations. A Mini Review

**DOI:** 10.3389/fmed.2021.639758

**Published:** 2021-04-09

**Authors:** Ina Kötter, Fabian Lötscher

**Affiliations:** ^1^Division of Rheumatology and Inflammatory Rheumatic Diseases, University Hospital Hamburg Eppendorf and Clinic for Rheumatology and Immunology Bad Bramstedt, Bad Bramstedt, Germany; ^2^Department of Rheumatology and Immunology, Inselspital Bern, University of Bern, Bern, Switzerland

**Keywords:** BS, manifestations, vascular, neurologic, musculoskeletal, gastrointestinal, cluster

## Abstract

Behçet‘s Syndrome (BS) is a variable vessel vasculitis according to the Chapel Hill Consensus Nomenclature (1) and may thus affect any organ, including major and minor arterial and venous vessels to a varying degree and with varying frequency. Although the main features of BS are recurrent oral and genital aphthous ulcers, cutaneous lesions, ocular inflammation and arthritis—major vessel and life—or organ threatening involvement of internal organs and the central and peripheral nervous system occur. In general, BS in Europe appears to form six phenotypes of clinical manifestations (2), which are (1) mucocutaneous only, (2) predominant arthritis/articular involvement, (3) vascular phenotype, (4) ocular manifestations, which are most likely associated with CNS manifestations and HLA-B51, (5) dominant parenchymal CNS manifestations (being associated with the ocular ones), and (6) gastrointestinal involvement. Mucocutaneous manifestations are present in almost all patients/all phenotypes. In the following review, we summarize the current knowledge concerning vascular, neurologic, gastrointestinal and musculoskeletal manifestations of the disease.

## Introduction

Behçet‘s Syndrome (BS) is a variable vessel vasculitis according to the Chapel Hill Consensus Nomenclature (1) and may thus affect any organ, including major and minor arterial and venous vessels to a varying degree and with varying frequency. Although the main features of BS are recurrent oral and genital aphthous ulcers, cutaneous lesions, ocular inflammation and arthritis—major vessel and life—or organ threatening involvement of internal organs and the central and peripheral nervous system occur. In general, BS in Europe appears to form six phenotypes of clinical manifestations (2), which are (1) mucocutaneous only, (2) predominant arthritis/articular involvement, (3) vascular phenotype, (4) ocular manifestations, which are most likely associated with CNS manifestations and HLA-B51, (5) dominant parenchymal CNS manifestations (being associated with the ocular ones), and (6) gastrointestinal involvement. Mucocutaneous manifestations are present in almost all patients/all phenotypes. A cluster analysis in China revealed 5 clusters, the most common being the mucocutaneous one, followed by the articular one, the gastrointestinal, the ocular, and the cardiovascular cluster. These were distributed differentially among male and female patients, with cluster 1 occurring predominantly in female patients, whereas there was a strong male predominance in the uveitis and cardiovascular cluster (number 4 and 5). All other clusters were distributed almost equally among both genders (3). A considerable number of reviews and good prospective clinical trials exist for the typical mucocutaneous and ocular manifestations of BS. In the following review, we will provide an overview of the less frequently documented manifestations of the disease, which can nevertheless be decisive in terms of prognosis and differential diagnosis.

## Vascular Involvement in BS

By definition, vessels of every size either arterial or venous can be affected by the vasculitis in BS. Venous vessels are affected much more frequently than arteries, which is unique among the vasculitides (4). Histopathologically, in the veins there is a scarce inflammatory infiltrate, fibrous thickening of the vessel wall and focal aneurysmal dilatation, the vessel being occluded by an organized thrombus (5). Thrombembolism is not a feature of venous thrombosis in BS. Many common and well-known prothrombotic factors such as factor V Leiden mutation, protein C or S deficiency, prothrombin fragments, etc. have been discussed in the pathogenesis of thrombosis in BS, but the data are conflicting. A recent meta-analysis on antiphospholipid antibodies revealed a significantly high prevalence of anticardiolipin (ACL) and anti-ß2-glycoprotein antibodies (6).

In the large arteries, inflammatory infiltrates mainly consisting of neutrophils, lymphocytes, and plasma cells in the adventitia and media, sometimes resembling granulomas in Takayasu‘s arteritis, were found in 8 cases from Japan. In the media, loss of elastic fibers and muscle fibers and proliferation of fibroblasts occurred. Intimal thickening of vasa vasorum was noted. Scarred arteritis of major aortic branches was noted in five of these patients, mainly affecting aneurysms which had formed (7).

The frequency of vascular manifestations of BS is estimated at 5–40% (4), depending on the population in which the evaluation was performed, and males are affected more often than females. The first manifestation occurs a median time of 5 years after primary diagnosis of BS in 75% of the patients.

### Venous Manifestations

Superficial venous thrombosis (SVT) followed by deep vein thrombosis (DVT) are the most frequent vascular manifestations. There is a predominance of young males, and the DVT in contrast to those of other origin tend to relapse and are often bilateral. They affect up to 13% of BS patients (8, 9) and may lead to post-thrombotic syndrome in the most severe cases (10, 11). Although thrombosis mainly occurs in the upper and lower limbs, also uncommon sites [such as superior or inferior vena cava, hepatic vein with Budd Chiari syndrome (BCS), portal vein, cerebral venous sinus, intracardiac thrombi in the right ventricle] may be involved (12–14). The occurrence of thrombosis at these unusual sites is relatively specific to BS (15).

Hence BCS, an occlusion of the intrahepatic veins is caused most frequently by BS in countries where BS is endemic. However, in BS itself it is rare with a frequency of <5% (16). In BS it is mostly accompanied by thrombosis of the vena cava inferior and often also by lower extremity DVT and has a high mortality rate as shown in Table 1 (4, 8, 13, 19–21).

**Table 1:** Overview of morbidity and mortality of different organ manifestations in BS.

	**Mortality**		**Morbidity**	**References**
	**Mortality rate (%)**	**Median follow up time (y)**		
**Behçet syndrome (all manifestations)**	5–10	7.7–20	Increased mortality in major vessel and CNS disease young (15–24 year) and male patients, with high number of disease flares with worse outcome and high unemployment and dependence rate	(17, 18)
**Vascular**	8	7.7	35.4% of patients with recurrent vascular events	(8, 18)
Venous	6–6.5	4.75–7.7		(18, 19)
Deep venous thrombosis (DVT)	3	4.75	Severe post-thrombotic syndrome (in 50% of patients) venous claudication (in 30% of patients) most common type of recurrent vascular manifestation	(8, 11, 19)
Thrombosis of vena cava	12	4.75		(19)
Budd Chiari syndrome (BCS)	18–47	4–9	Concomitant inferior vena cava thrombosis is common sequelae: portal hypertension, liver cirrhosis, hepatic failure, lower extremity edema poor prognosis in liver failure	(13, 19–21)
Arterial	13–14	7.7	45.6% undergo surgery of which 34% had surgical complications (mainly prosthetic thrombosis, less frequent if immunosuppressants are applied)	(18, 22)
Pulmonary artery aneurysm (PAA)	26–50	1–7	Anticoagulation can worsen hemoptysis frequent thrombus formation within PAA poor prognosis with highest mortality rate in BS	(23–26)
Pulmonary artery thrombosis (PAT)	23	7	PAT can transform/progress into PAA pulmonary artery hypertension (in up to 50% of patients)	(23, 24)
Extrapulmonary arteries	4–17	4	High frequency of new aneurysms frequent graft obstruction (well-tolerated due to collateral formation)	(27, 28)
Cardiac^*^	15–28	3	Poor prognosis in coronary artery involvement (reduced cardiac function in 2/3 of patients) frequent relapse of pericarditis	(12, 29)
**CNS**	7–12	7.7–20	Worse prognosis in parenchymal involvement and abnormal CSF findings approximately 50% of patients with moderate to severe disability by 10 years	(17, 18, 30–32)
Parenchymal	11–21	4–20	44% one attack and remission 28% attacks with secondary progression 10% primary progression 21% silent neurological involvement 25–33.9% disabled or dead factors associated with poor outcome (disability or death): baseline hemiparesis or paraparesis and brainstem involvement	(17, 30–32)
Vascular	7	4.75	Good short-term outcomes predominant sequel: optic nerve atrophy/reduced visual acuity	(19, 33)
**Gastrointestinal**	2–5	5–7.5	Cumulative operation rate (5 years) 32% remission or mild clinical activity 72% multiple relapses/chronic symptoms 28%	(34, 35)
**Musculoskeletal**	6	7.7	Mainly non-erosive, but impact on quality of life	(18, 36, 37)

* *Pericarditis, valve insufficiency, coronary artery involvement*.

Interestingly, it could be shown that the thickness of the femoral vein wall measured with ultrasound is increased in patients with BS compared to healthy controls. This measurement may even facilitate the differentiation between inflammatory bowel disease and gastrointestinal involvement of BS. It may hint at a subclinical involvement of major vessels (veins) in all patients with BS independently of the clinical phenotype and may represent a new diagnostic tool (38, 39).

### Arterial Manifestations

Peripheral arterial manifestations are much less common than the venous ones. Their frequency is estimated at <5% (8, 22). They occur late in the course of the disease, 5–10 years after the first symptoms of BS (8). The majority present as aneurysms rather than with thrombi. Claudication of the affected limb or digital ulcerations and necroses are the main symptoms. The thoracic and abdominal aorta may also be affected, the abdominal aorta being the artery which is most commonly aneurysmatic (60%) (27, 28).

**Figure 1:**
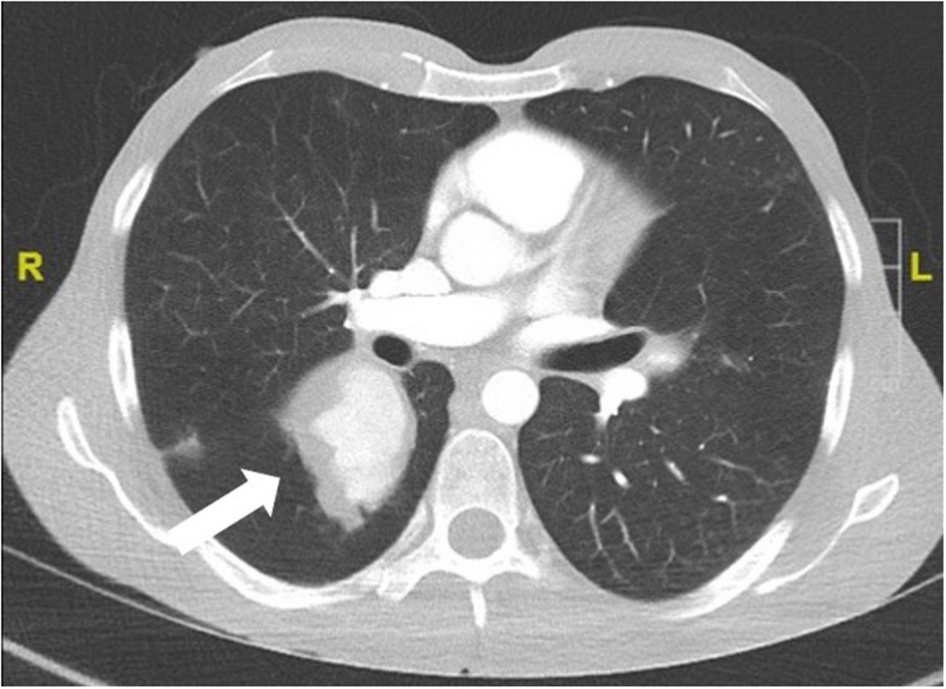
Pulmonary arterial aneurysm (CT scan with contrast agent) of a 24 year old male patient with definite BS and hemoptysis.

Pulmonary artery involvement has a prevalence lower than 5%. However, it is the most common form of arterial involvement. It occurs early in the disease course and in ~80% of the cases DVT of the extremities is present in parallel or has occurred 2–3 years before the involvement of the pulmonary artery. It manifests as pulmonary arterial aneurysms with hemoptysis and bilateral hilar opacities on imaging (Figure 1). Pulmonary artery thrombosis is present in one third of the patients with pulmonary artery aneurysms, and mild pulmonary artery hypertension may occur (23).

Rarely, coronary arteries with the formation of giant or multiple aneurysms or pseudoaneurysms, thrombi and occlusions/stenosis are involved, causing the symptomatology of a myocardial ischemia or infarction in a young patient. Mostly, the coronary artery involvement is accompanied by other vascular manifestations such as aortic or other arterial aneurysms or DVT. Interestingly, catheterization leads to aneurysm formation in the punctured vessels comparable to a “vascular pathergy phenomenon” (40, 41). In addition to the above-mentioned intracardiac thrombi and coronary vessel affections, further cardiac manifestations are pericarditis and endocardial involvement with valve insufficiencies (29).

### Coexistence of Venous and Arterial Involvement

Within the vascular phenotype of BS, several associations of vascular manifestations clustering together have been described (4), encompassing different combinations of vasculitic venous or arterial manifestations.

Significant correlations exist between cerebral venous thrombosis and pulmonary artery involvement (24), BCS and vena cava inferior syndrome. Lower (and, more rarely upper) extremity vein thrombosis is often present in all of these and may even precede them.

A special subtype of vascular involvement in BS is the Hughes-Stovin syndrome (HSS). This is the combination of deep vein thrombosis and pulmonary arterial aneurysms. Not uncommonly an intracardiac thrombus is also present, which was also the case in the three of the four patients Hughes and Stovin (42). To date, it is hypothesized that all patients with HSS have BS and that isolated HSS without any other BS symptoms is incomplete BS (43, 44).

### Disease Course and Prognosis

Vascular involvement is often accompanied by systemic symptoms such as fever (45). Increased serological markers of inflammation such as ESR and CRP are present. It has a relapsing course, and the relapses may occur anywhere, although they tend to occur more often in the same segment or in close proximity to it (4, 22).

Vascular involvement, especially of the arteries and large veins (BCS, vena cava), causes severe morbidity and increases mortality (17–19, 21, 25, 26) (Table 1).

## Neurologic Involvement in BS

Neurologic involvement, also called Neuro-Behcet‘s syndrome (NBS) occurs with a frequency of 3–30% (46). Males are more frequently affected than females, and it occurs a median time of 6 years after the first manifestations of BS (47). However, it can also be the first manifestation of BS, which in the cohort from Turkey was the case in 29% (47).

NBS is divided into a parenchymal form (over 80% of all NBD cases) and a vascular form (~20%). Both forms supposedly never occur simultaneously in one patient.

Peripheral nervous system (PNS) manifestations are very rare [3.9% of all NBS cases (47)], they include sensorimotor neuropathy, mononeuritis multiplex, and autonomic neuropathy, but also Guillain-Barré syndrome. Isolated cranial nerve neuropathies exist and are associated with signs of inflammation on lumbar puncture. Optic neuropathy is rare in the absence of panuveitis with retinal vasculitis (48). The peripheral nervous system manifestations can be associated with both parenchymal and vascular NBS manifestations.

### Parenchymal CNS Manifestations

Parenchymal NBS (pNBS) mainly occur in the brain, but the spinal cord may rarely (in up to 10% of NBS in large series) also be affected in the form of a transverse myelitis (49). In the latter case, the leading clinical symptom is sensory disturbance, weakness, sphincter or sexual dysfunction, depending on the localization of the inflammatory lesions.

**Figure 2:**
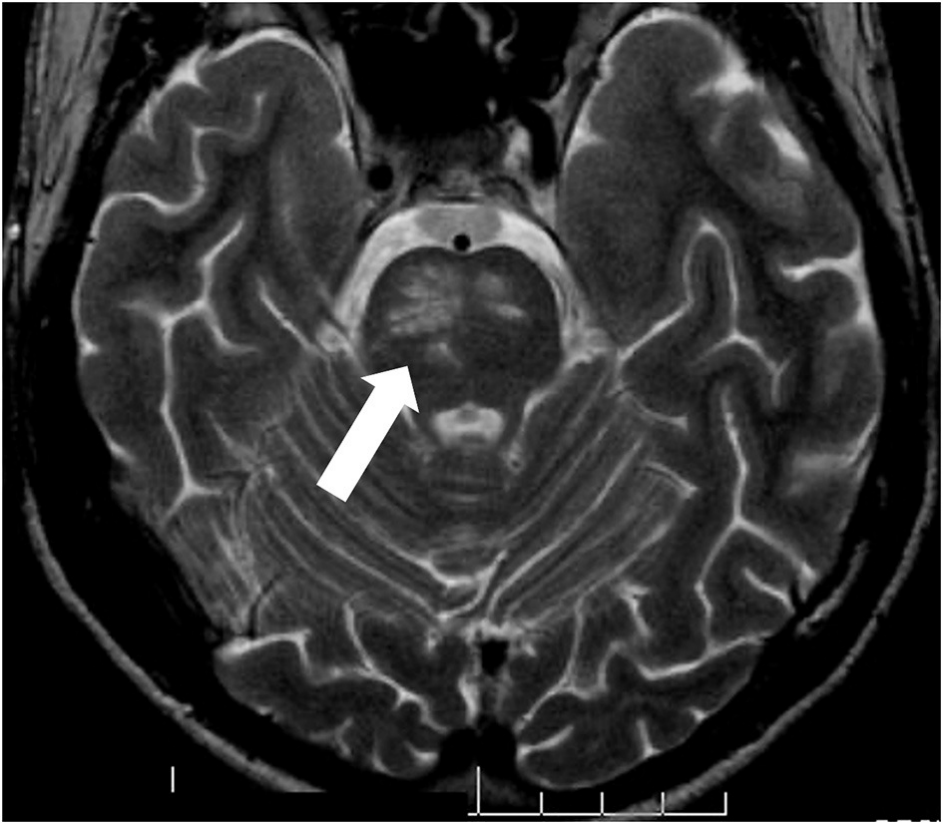
pNBS with a vasulitic lesion in the brainstem (MRI FLAIR sequence). Twenty eight years old male patient with aphasia, hemiparesis, who had definite BS according to the international study group criteria since the age of 21.

In 50% of the parenchymal CNS manifestations the brain stem is affected, where inflammatory lesions can be found on MRI (Figure 2). Characteristic is a lesion in the midbrain or pons (30, 31). The clinical symptoms of these patients are primarily systemic such as fever and fatigue, followed by severe headache, and then after a few days focal neurological signs develop. These often consist of ophthalmoparesis in combination with ataxia and asymmetric long tract signs.

Another pattern of pNBS on MRI is a more diffuse distribution of lesions in both hemispheres, clinically presenting with progressive encephalopathy consisting of headaches, focal signs such as dysphasia, hemiparesis, sensory loss, and cognitive impairment.

Rarely, so-called “tumor-like lesions” are seen on MRI, a large mass develops in one hemisphere, mimicking glioma, or lymphoma. These lesions are frequently biopsied and resolve with treatment, but they often leave neurological sequelae (50).

#### Laboratory and Histopathological Findings in pNBS

In the cerebrospinal fluid (CSF), in two thirds of pNBS increased proteins and/or pleocytosis are found, in 54% with neutrophil predominance or a mixture of neutrophils and lymphocytes, 46% are purely lymphocytic. Oligoclonal bands are found in 16%. In one third the CSF may be completely normal in spite of typical pNBS (30).

Histopathologically, either multifocal neutrophilic perivascular inflammation (51) or perivascular mononuclear cellular infiltrates were found, the latter consisting of CD45RO+ T-Lymphocytes and CD68+ monocytes with a few CD20+ B-lymphocytes. In long-term remission, gliosis, and atrophy of the affected areas were shown, with some viable neurons. There were scattered infiltrates and foci of perivascular inflammation in a Japanese case series of 3 patients with NBD who were either diagnostically biopsied or histopathology was obtained upon autopsy (52).

### Vascular Nbs (vNBS)

VNBS almost exclusively consists of cerebral venous thrombosis of the superior sagittal or transverse sinus and accounts for up to 20% of all NBS cases (33). There is an association with systemic vascular manifestations of BS (53).

Clinical symptoms are severe headache, evolving over a few weeks, and upon neurological examination papilledema, and occasionally sixth nerve palsy are revealed. MRI usually shows an occluded dural sinus, venous infarctions are rare. CSF findings are usually normal, except an increased pressure.

### Diagnosis of NBS

The diagnosis of NBS should be made according to the International Consensus Recommendation (ICR) criteria (54). They can be summarized as “the occurrence of neurological signs and symptoms in a patient that meets the ISG criteria for BS that are not otherwise explained by any other known systemic or neurological disease or treatment and in whom objective abnormalities consistent with NBS are detected either on neurological examination, neuroimaging studies, magnetic resonance imaging (MRI), or abnormal CSF examinations.” However, patients in whom NBS is the first manifestation of BS, and who do not fulfill the diagnostic or classification criteria for BS, diagnosis is very challenging.

The differential diagnosis from multiple sclerosis (MS) may be difficult, as clinical symptoms, as well as MRI lesions and even inflammatory markers in cerebrospinal fluids may be very similar, even oligoclonal bands in CSF occur in MS as well as in NBS. Hence, there were attempts to find either biomarkers in CSF (55–57) or even typical signs of vasculitis of BS in brain biopsies (58). One study suggests specific features of BS on MRI, such as a higher frequency of periventricular lesions in MS than in inflammatory vasculopathies, and the highest frequency of perivenular lesions (34%) in pNBS also in comparison to other CNS vasculitides (59).

### Disease Course and Prognosis

For pNBS different courses are described: Single attack, relapsing, secondary progressive, primary progressive (60). In a French cohort with 115 patients, 68% had acute forms of NBS, and 32% a progressive form. 40% of the patients had severe disability at baseline, the 5- and 7-year event-free survival rates were 65 and 53%. After a median follow-up of 73 months 25.2% of the patients became dependent, which means they were unable to perform activities of daily living or died. Factors independently associated with poor outcome were paresis at onset (OR 6.47), and location of inflammatory lesions at the brainstem on MRI (OR 8.41) (32) (Table 1).

There appears to be an association with ocular involvement and with HLA-B51 positivity, the latter also indicating a worse prognosis (61) and also being associated with an increased relapse risk (OR 3.5) in the French cohort.

## Gastrointestinal Manifestations of BS (GIBD)

Symptoms (such as abdominal pain) indicating gastrointestinal manifestation are seen in up to 50% of patients with BS, especially in the Far East (62). However, the prevalence of endoscopically confirmed cases is generally much lower, but still shows a clear geographic gradient: while the prevalence in Japan or Korea is reported in 3.2–37% of patients, the prevalence in Turkey is only just under 1–5% (34). Care should be taken when interpreting and comparing the different data: on the one hand, the methods of diagnosis (symptom-based vs. endoscopy) differed, on the other hand, various classification criteria were applied. GIBD seems to occur some years after the first manifestation of oral ulcers, there is no gender difference (63).

### Clinical Manifestation and Laboratory Findings

GIBD presents with a broad range of symptoms, dependent on the anatomic localization of involvement. Typically, affected patients suffer from abdominal pain. More than 25% of patients complain about diarrhea and/or GI-bleeding, rarely vomiting, and weight loss are reported (64, 65).

There are no specific laboratory tests reflecting GI-involvement in BS. Fecal calprotectin seems to be a promising tool for non-invasive detection of intestinal involvement and could also be used to monitor intestinal disease activity in the future (66, 67). A recent meta-analysis underscores the strong association of serum anti-Saccharomyces cervisiae antibodies (ASCA) with GIBD (68). But ASCA-positivity has only limited diagnostic value in GIBD, as the antibodies are frequently found in Crohn's disease (CD), the main clinical differential diagnosis of GIBD.

### Endoscopic Findings and Histology

**Figure 3:**
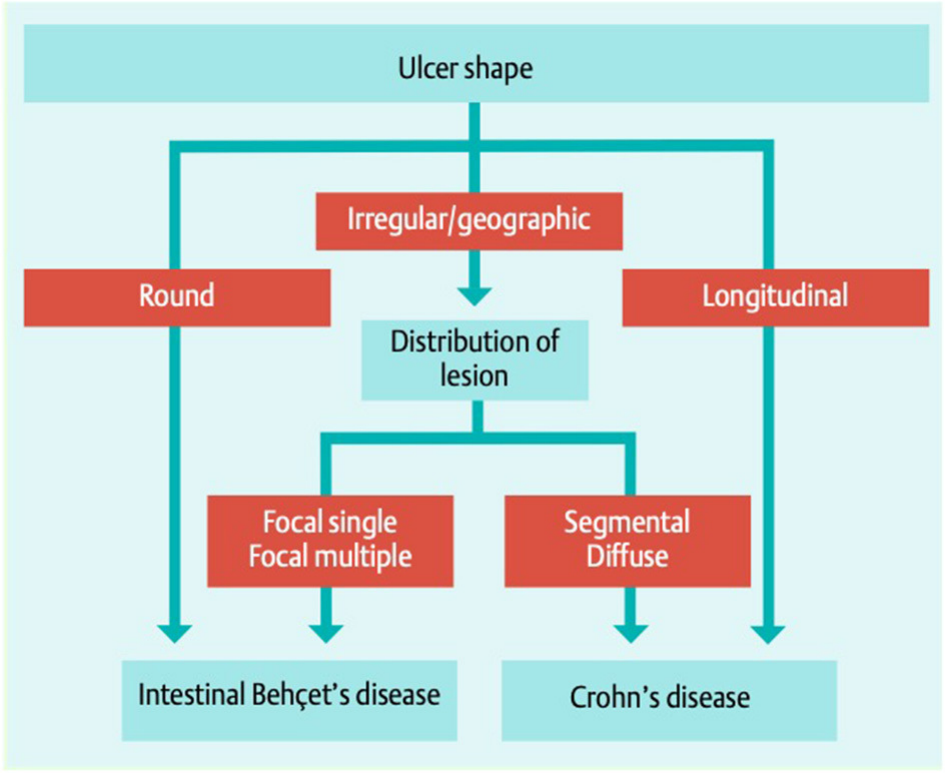
Simple decision tree of colonoscopic findings, distinguishing GIBD from CD Lee et al. (71). With permission of the author and the publisher.

Colonoscopy remains the examination method of choice and in the majority of subjects with GIBD characteristic mucosal lesions can be found in the terminal ileum and the area of the coecum (64), but the disease can manifest along the entire gastrointestinal tract, including extra intestinal organs (pancreas, liver, and spleen). Typical ulcers are large (>1 cm) and deep with round or oval shape and appear localized, single, or in small groups (up to 5). Multi-segmental or diffuse distribution of the lesions are hardly seen (in 2 and 4%, respectively), as well as ano-rectal involvement (69). CD and GIBD share similar extraintestinal features and endoscopic differentiation is challenging (70). Lee et al. proposed a diagnostic algorithm to separate the two manifestations, shown in Figure 3 (71). The same group proposed diagnostic criteria for GIBD, validated in a Korean population (72). In many cases, histological processing of the samples taken does not provide any clarification: while a lymphoplasmocytic infiltration with sometimes vasculitic changes is usually observed in GIBS, granulomatous changes rather indicate the presence of a CD (73). The latter, however, are only described in 15–36% of patients with CD (66), and are occasionally also observed in GIBS (74). Other important differential diagnoses apart from CD are: intestinal tuberculosis (especially in endemic areas), ulcerative colitis and NSAID induced colitis (34).

Gastroduodenal or esophageal involvement have been reported to be rare, the latter being more common in men (75). However, due to the infrequency of gastroduodenoscopy in BS and a lack of large studies, underreporting of these manifestations might be possible (76). In cases of abdominal complaints, elevated inflammatory markers (as CRP or fecal calprotectin) with normal conventional endoscopic examinations, capsule endoscopy (CE) can be used to reveal pathologies of the small intestines (34, 77).

### Disease Course

Although more than 50% of Patients with GIBD have a rather mild disease course, intestinal manifestations bear the risk of life-threatening manifestations with complications such as intestinal perforation, perianal, or entero-enteric fistulae as well as intestinal bleeding, requiring surgical intervention in up to 1/3 of all patients (35) (Table 1).

## Musculoskeletal Involvement in BS

Musculoskeletal involvement in BS is common, the frequency of joint involvement varies considerably depending on geographic location (36) and study design as well as the evaluating medical subspeciality and the definition of involvement (arthritis vs. arthropathy). Prevalence ranges from 39 to 70%, mostly around 50% (36, 78–80).

### Characteristics of Joint Involvement

According to the largest prospective studies (36, 37, 78, 79) arthritis in BS usually affects large peripheral joints, in decreasing frequency knees, ankle joints, wrists, hands, and rarely elbows, shoulders and feet, as shown in Table 2 (36, 78, 79, 82). The involvement is primarily mono- or oligoarticular, data concerning symmetry of joint distribution are conflicting: in some studies with a tendency to symmetric distribution if the manifestation is not monoarticular (79), favoring asymmetric involvement in others (37). Generally, polyarticular manifestation is rare in BS (36, 79). Some smaller studies observed a polyarticular course in the majority of cases (82). The arthritis in BS is recurrent in nature, with acute and self-limiting course, mostly of short duration (2 weeks to 2 months) but chronic courses were rarely observed (36, 78, 79). Radiographic evaluation is usually inconspicuous, although sporadic cases of joint erosion have been reported (83, 84).

**Table 2:** Characteristics of arthritis in BS.

Localization^*^	Knee	33%
	Ankle joint	24
	Wrist	14
	Hand	11
	Foot	8
	Elbow	5
	Sacroiliac joint	<5
	Hip	<5
	Shoulder	<5
Number of joints^†^	Monoarticular	66
	Oligoarticular	29
	Polyarticular	4
Duration^†^	Acute (<6–8 weeks)	89
	Chronic (>8 weeks)	11
Synovial fluid examination^§^	Cell count	11,000/ul (1,600–36,000/ul)
	Cell type	Predominantly polymorphonuclear
Radiography	Typically non-erosive	

* *Calculated with a total of 609 arthritis episodes extracted from the publications of Yurdakul et al. (79), Gur et al. (37), and Fatemi et al. (36)*.

† *Calculated with a total of 493 (duration) and 369 (number of Joints) arthritis episodes extracted from publications of Yurdakul et al. (79) and Fatemi et al. (36)*.

§ *Calculated with extraction 38 synovial fluid analyses from Yurdakul et al. (79) and Gibson et al. (81)*.

Several studies report an association of arthritis with the extra-articular manifestation papulo-pustulosis, implying this form of disease-manifestation being a cluster of disease expression (36, 85–87). Furthermore, Hatemi et al. observed that in BS patients with arthritis and acne, enthesopathy (including enthesitis) is more commonly encountered on sonography than in patients without arthritis (88). The first finding raises the question of a possible pathogenetic connection of BS to acne associated arthritis syndromes. There was a long debate about the inclusion of BS in the group of seronegative spondylarthritis (SpA) (89), as some investigators reported a high prevalence of sacroiliitis in small cohorts (90). Prospective studies have not yet been able to substantiate these observations (79, 91–93).

Although SpA and BS share many clinical features (such as skin, joint, eye, and GI manifestations), the rarity of axial involvement in BS has led to the omission of BS from the SpA group. Nevertheless, SpA and BS (as well as psoriasis) share HLA class 1 association, which is a strong common immunopathogenetic feature. Furthermore, these diseases have the commonality of barrier dysfunction (oral mucosa, gut, and skin) as well as aberrant immune reactions at sites of physical stress (entheses, mini-entheses in the eye, vessel walls). These common features led to the recently established concept of, MHC-I opathies' (94). However, we have to take into account that on the one hand, only a part of the patients is HLA-B^*^51 positive, on the other hand less frequent manifestations like CNS involvement and vasculitis do not seem to be HLA-associated, which implies a multifactorial and/or sequential disease genesis which is discussed elsewhere (95).

### Laboratory Findings, Synovial Fluid Characteristics, and Synovial Histology

Acute phase reactants (ESR, CRP) are usually elevated in patients with arthritic episodes, antibody tests (rheumatoid factor, ACPA, and ANA) are negative (37, 79, 82). Synovial fluid analysis typically reveals mild, unspecific inflammatory changes with predominantly polymorphonuclear leucocytes (79). Synovial fluid cell counts range between 1,600 and 36,000/ul (Table 2) (79, 81), with normal glucose levels and elevated complement C3 and C4 levels (96). Pay et al. found synovial pro-inflammatory cytokines IL-18, TNF-alpha, and MMP-3 levels to be lower in BS than in RA patients but found IL-1 levels to be elevated (97).

Synovial histopathology was only investigated in small studies, examinations mainly show non-specific inflammatory changes. In 1978 Vernon-Roberts et al. described replacement of the superficial zone of the synovium by heavily inflamed granulation tissue, similar findings were confirmed by Yurdakul et al. in (79) and Vernon Roberts et al. (84). Synovitis in BS is characterized by polymorphonuclear neutrophil and T-cell predominance, without signs of neutrophil vasculitis (98).

### Quality of Life and Fibromyalgia

A small study by Gur et al. in 2006 showed that arthritis in BS negatively affects quality of life (in comparison to healthy subjects and BS patients without arthritis), with equal anxiety and depression levels (37). Limited evidence suggests that there is increased prevalence (5.7–37%) of fibromyalgia in BS patients compared with the general population (2.9–4.7%) (99). Consistent with reports in the general population, fibromyalgia in BS patients is more frequent in female patients.

### Further Locomotor Involvement

Few cases of necrotizing myositis in BS have been reported (100), an both focal and generalized manifestations have been described. Osteonecrosis is rarely observed in patients with BS and could be corticosteroid-associated in a significant proportion of cases described (101).

## Summary and Perspective

Vascular and neurological manifestations of BS are relatively rare but are associated with a high morbidity and mortality. This is especially true for the arterial vascular manifestations and parenchymal neurological manifestations involving the brainstem. Gastrointestinal involvement is very rare but is also associated with an increased morbidity. Even the much more common articular/locomotor involvement impairs quality of health and life of the patients with BS. A limitation of this mini review is, that there are only a few and mostly retrospective studies on the manifestations of BS covered here and the methods as well as evaluation criteria were not uniform. Furthermore, the cohorts published so far were quite small and mostly from a single specialized center. In the future, international registries may be helpful to collect data in larger, multicenter cohorts and the new OMERACT outcome measures will standardize and simplify the evaluation of the course of the diverse manifestations of BS (102).

## Author Contributions

IK: wrote the part on vascular and neurologic manifestations. FL: wrote the part on articular and gastrointestinal manifestations. Both authors contributed to the article and approved the submitted version.

## Conflict of Interest

The authors declare that the research was conducted in the absence of any commercial or financial relationships that could be construed as a potential conflict of interest.
